# The Appointments

**Published:** 1895-01

**Authors:** 


### THE APPOINTMENTS.

Governor Culberson has done a most sensible thing—one which will be gratefully remembered too by the people of Texas, in reappointing Dr. R. M. Swearingen State Health Officer. This appointment is a conspicuous exception to the policy introduced by the Governor-elect,—rotation in office,—for in nearly every other instance a new man has been appointed to the head of the departments.

Dr. Swearingen's host of friends throughout the State will rejoice with us at the announcement; and for two years more—four we hope—the people can rest in peaceful security, so far as any fears of epidemic disease might tend to disturb them, in the knowledge that the "Captain is on deck still,"—he who for so many years has successfully guarded the public health with sleepless vigilance. His re-appointment is a deserved compliment. It is a position hard to fill; one which, indeed few could fill satisfactorily. Indeed, under the law, it is an office to which few are eligible. First, the appointee must be a skilled physician, who has had active experience in the treatment of yellow fever, which presupposes that he must, himself, have had the fever,—otherwise he would constitute in his own person a large element of danger, for he must board and go through every infected ship that arrives in any Texas port; and he must also be "pledged to the necessity and importance of both sanitation and hygiene." So,—there are, indeed, few doctors in Texas, who come strictly within the meaning and intent of the law.

Dr. Swearingen has been State Health Officer ten years, and what he didn't know about its duties he has acquired by experience. As his administration has, every year, been characterized by the successful exclusion of foreign infection, and the prompt suppression of that of local origin,—by harmony and economy,—there would not have been a shadow of excuse for substituting a new man in his place; and we congratulate the Governor upon the wisdom and discretion of his choice, the Doctor upon his good fortune, and the people of Texas upon this guarantee of future safety. Especially are the Doctor's Austin friends proud of his election, and the JOURNAL extends to him hearty congratulations.

The Governor has not shown the same wisdom and sagacity, we fear, in some of his other appointments; though, so far as we know, he has in every instance appointed those he believed to be good men; at least, men who came to him highly endorsed. The sentiments of the TEXAS MEDICAL JOURNAL are so well known upon this subject that it is not necessary to say more than that we would have preferred to see Drs. White and Preston reappointed to the asylums, feeling that the best interests of the State and the insane are not promoted by changes in the managment, especially, when it has been harmonious and satisfactory, and characterized by economy and success. As we pointed out last month, Dr. White, who has had a longer experience with the insane than any man in Texas, showed in his report the lowest death rate and the smallest *per capita* expense since the founding of the asylum; and Dr. Preston's report made nearly as good a showing. However, Governor Culberson had his reasons for the changes; they were satisfactory to him, and we have no further criticism to make.

Dr. C. T. Simpson, of Temple, has been appointed Superintendent of the Austin Insane Asylum, vice Dr. F. S. White, whose time expired with the outgoing administration. The JOURNAL knows nothing of Dr. Simpson, nor of his qualifications for this responsible trust—further than that he is a respectable physician of Bell county—a practitioner of some twenty years or more. He has our best wishes for success. Dr. White leaves the institute in first-class condition, in smooth-running order, and we hope his successor may have smooth sailing. He has selected, we learn, Dr. T. O. Maxwell for his first assistant. Dr. Maxwell has held the position of second assistant physician under Dr. White for the past four years, and should be of great assistance to the new Superintendent till he "learns the ropes." We are not informed at this writing who will succeed Dr. Maxwell as second assistant; there are several applicants.

Dr. B. M. Worsham, of Ellis county, who, for four years, has been first assistant physician at the Austin Insane Asylum, has been appointed Superintendent of the Southwest Texas (San Antonio) Insane Asylum, vice Dr. Barker, who goes out with the old administration; and Dr. C. M. Rosser, of Dallas, succeeds Dr. John Preston as Superintendent of the (Terrell) North Texas Insane Asylum. The JOURNAL is not prepared to express an opinion upon the wisdom of this selection, nor upon the doctor's qualification and fitness for the position. Dr. Rosser is an active young practitioner, very ambitious of distinction, and no doubt will readily adapt himself to, and qualify himself for his responsible new duties. His assistants have not yet been announced, nor have those of Superintendent Worsham.

We learn that Dr. White, the retiring Superintendent of the Austin Asylum, will remove to Houston and engage in general practice. Wherever he goes he carries with him the best wishes of the JOURNAL and the people of Austin generally, in whose heart he and his interesting little family have a warm place. Apropos, the employes of the asylum, upon his retirement from the Superintendency, presented him with a handsome sterling silver tea service as a testimonial of their affection, regard and esteem.

There is some talk of Dr. White's establishing a private insane asylum in Texas, but of this, as yet, we are not prepared to speak. That there is an opening for something of the kind would seem to be demonstrated by the fact that there are far more insane in Texas than can be accommodated in the asylums; and as preference is given by law to the indigent over the pay patients, and therefore a large number of applicants who are well able to pay, have been necessarily refused admission in the State Asylums, it would seem that a private institution for such and kindred cases, would not want for patronage, and could be filled by Texas patients alone. It is estimated by both Dr. White and Dr. Preston that there are over one thousand insane in Texas outside of the asylums; of these, it is to be presumed, a large number would be able to pay for treatment. We hope to be able to have something further to say on this subject by and by.

Dr. Preston, the retiring Superintendent of the Terrell Asylum, we learn, will return to San Antonio and resume general practice there. At the time of his appointment, four years ago, he was doing a large practice there.

Dr. Becton, the new Superintendent of the State Institution for the Blind, who succeeds Dr. Rainey—(who may be said to be the father of the institution, he having been twenty years consecutively in charge,)—is too well known throughout Texas to require any commendation at our hands. The State papers have been very complimentary to the Doctor in speaking of his appoinment, which is very gratifying to his many friends.

P. S.—Dr. T. C. Karnes, of Gonzales, has been named for assistant physician at the Southwestern Asylum, San Antonio.

